# Hematemesis: An Exceptional Method of Revealing Gastric Metastasis From an Unknown Breast Cancer

**DOI:** 10.7759/cureus.18987

**Published:** 2021-10-23

**Authors:** Rachid Jabi, Nassira Karich, Mouad Ouryemchi, Amal Bennani, Mohammed Bouziane

**Affiliations:** 1 Department of General Surgery, Mohamed VI University Hospital, Faculty of Medicine and Pharmacy, Labarotory of Anatomy, Microsurgery and Surgery Experimental and Medical Simulation, Mohammed First University, Oujda, MAR; 2 Department of Anatomopathology, Mohammed VI University Hospital, Faculty of Medicine and Pharmacy, Oujda, MAR

**Keywords:** chemotherapy, case report, gastric metastasis, hematemesis, breast cancer

## Abstract

Breast cancer is a public health problem with an annual incidence exceeding two million new cases leading to its classification as first cancer in women. Its clinical presentation is variable and the palpation of a breast nodule allows the diagnosis to be suspected in the majority of cases. The treatment is multidisciplinary and has health, economic, and psychological impacts.

We report a sporadic case of a 60-year-old woman followed for epigastralgia for three months. The current history of the present disease goes back to three days before her admission with the onset of several episodes of hematemesis motivating the patient to consult the ER and then get admitted to the ER for hematemesis. The initial clinical examination showed an epigastric sensitivity in a hemodynamically stable patient. The endoscopy revealed a gastric tumor and immunohistochemistry confirmed the gastric localization from a breast cancer unrecognized by the patient and discovered at the careful clinical examination after having stabilized the patient's condition.

## Introduction

Breast cancer is the second most common cancer in terms of incidence and mortality in the world, with an estimated 2.088 million new cases of which 11747 cases are recorded in our country [1]. Despite the standardization of screening strategies [2] and the advent of immunotherapy [3], the prognosis of advanced and metastatic forms remains poor [4]. We describe, according to CAse REport (CARE) guidelines, an exceptional case of breast cancer unrecognized by a patient and discovered on a gastric metastasis following the endoscopic exploration of a digestive hemorrhage [5].

We hope through this case presentation to enrich the literature published on this subject and highlight the primordial place of screening and systematic clinical examination of the breasts once a woman consults for any medical condition.

## Case presentation

**Figure 1:**
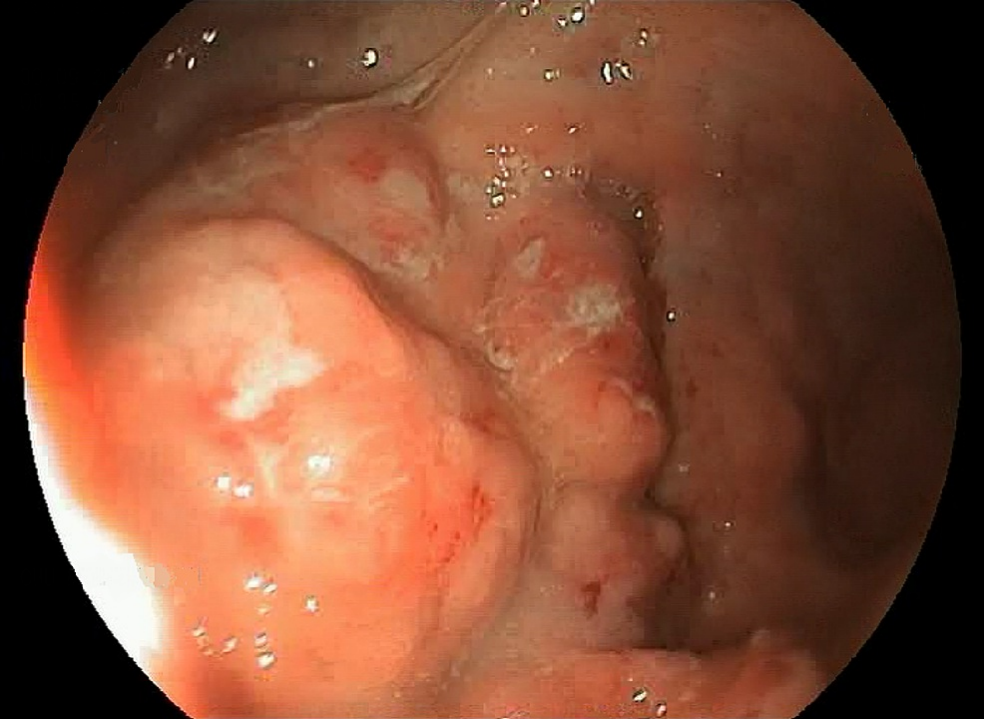
Endoscopy showed a bleeding gastric process with biopsy.

The patient was a 60-year-old woman from the north-eastern part of Morocco, a nursing mother of three children, who had been taking oral contraception for 30 years and had been followed up for epigastralgia for three months. The history of the present disease goes back to three days before her admission with the onset of several episodes of hematemesis motivating the patient to consult the ER. The rapid clinical examination performed in the ER revealed an epigastric sensitivity in a patient who was hemodynamically and respiratorily stable. The biological analyses performed in the ER to evaluate the biological impact of the bleeding showed profound anemia with Hb at 8.1 g/dl, a transfusion was initiated and then the endoscopy showed a bleeding gastric process with biopsy for histological study (Figure 1).

**Figure 2:**
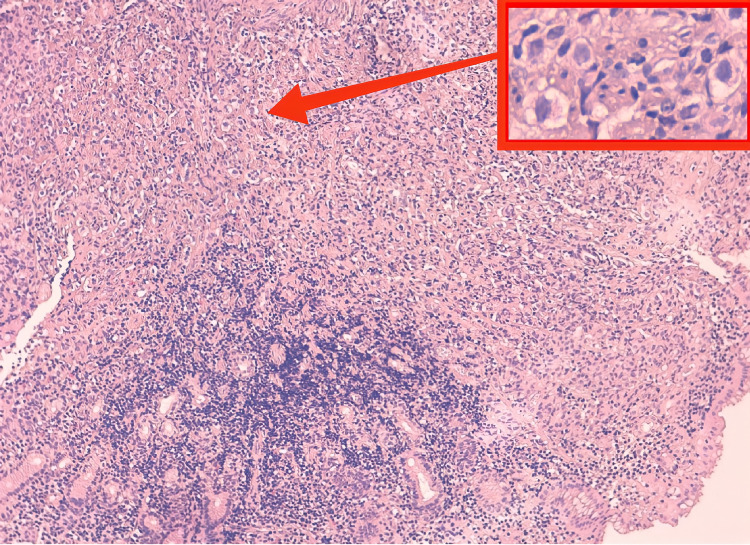
Invasive breast carcinoma with signet ring cells features.

**Figure 3:**
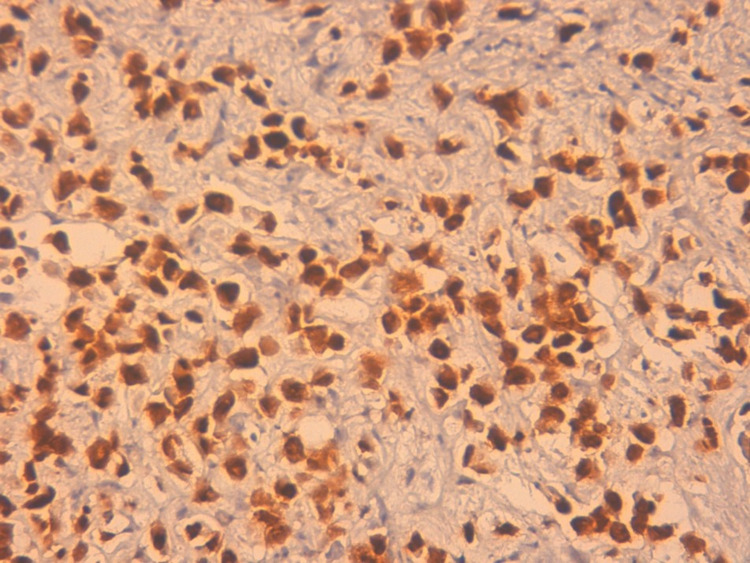
Immunohistochemical staining of GATA3 confirmed its breast origin ( field x40).

**Figure 4:**
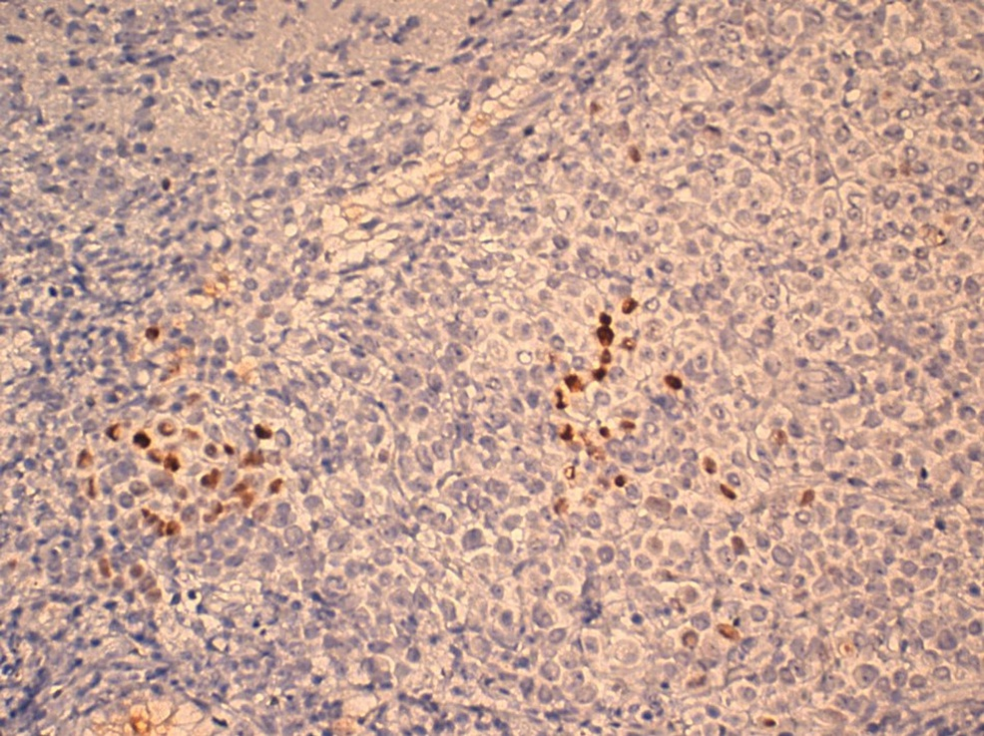
Progesterone receptor, weak focal staining (field x20).

**Figure 5:**
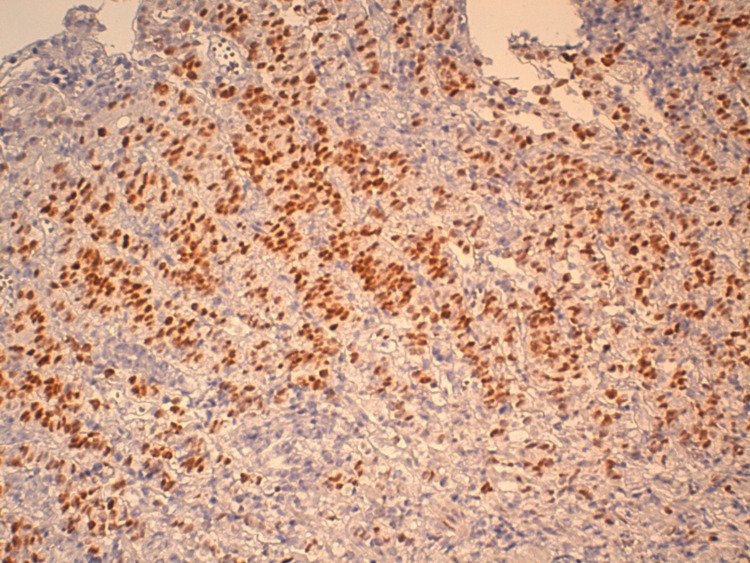
Estrogen receptor, uniform strong staining (field x20).

Up to this stage, the care team monitored the hemodynamic status of the patient who was receiving the proton pump inhibitor (PPI) by injection, expecting an adenocarcinoma on the biopsy but the anatomopathological results were in favor of invasive breast carcinoma with signet ring cells features (Figure 2) confirmed by an immunohistochemical study (Figures 3-4-5). In front of this very rare case and after a multidisciplinary discussion, we re-interviewed the patient who had forgotten to report the notion of simple breast pain for three months. Moreover, careful palpation of the right breast had revealed a small nodule of less than 1 cm mobile located in retro nipple without axillary adenopathy whose ultrasound-mammography was in favor of a breast node classified BIRADS 4. Consequently, we performed a breast biopsy in favor of lobular type cancer and a radiological extension study without any other secondary location except the stomach; classifying our patient as T1NxM1. A decision to put the patient on palliative chemotherapy was the conclusion of the multidisciplinary discussion.

In the follow-up of our patient, she presented febrile neutropenia as a side effect of the first chemotherapy treatment, the management of which was complicated by a respiratory infection of the COVID-19 type leading to her death a few days later.

## Discussion

According to the latest National Comprehensive Cancer Network (NCCN) report, breast cancer is first cancer diagnosed in women and the second leading cause of cancer death [1], while gastric cancer is the fifth most common cause of death [6].

Thanks to the guidelines of the Breast Health Global Initiative, screening strategies have improved the prognosis of this deadly cancer [2], whose prognosis in metastatic forms remains poor [7] despite the advent of several new therapeutic means including immunotherapy [3].

Its metastases classify the disease in stage IV [8] and can be revealing of cancer in the absence of the tumor syndrome at the breast level in certain cases. Thus, the highest incidence of breast metastases in the gastric region did not exceed 0.3% with cases discovered on autopsy specimens [9].

In our patient, the symptomatology of discovery was a digestive hemorrhage on gastric metastases without self-palpation of a nodule by the patient. In the same context, the annual palpation of the breasts is a sacred right for all women over 25 years of age and the radiological screening examination is essential from the age of 50 years onwards, which all health practitioners must commit to and validate, regardless of the clinical presentation of the patients of female sex [2].

Throughout the literature, the GI tract was rarely a preferred metastatic site for breast cancer in particular [10]. Thus Apodaca-Rueda M et al had reported a case of metastasis to the pancreatic level [11], Nour A et al., described a metastasis to both the colonic and gastric level initially taken as primary [12]. Other authors report digestive metastases of a breast primary discovered in a context of complication or emergencies like Théraux J et al. who had described a colonic occlusion on a colonic tumor of breast origin [13] and Nehmeh WA et al. had reported a secondary localization following a gastric perforation [14], while hematemesis revealed the diagnosis in our case. This sporadic case is described among less than 50 cases reported through the literature [14] and the treatment of its rare forms of GI metastases of breast origin is based on palliative chemotherapy [10] with a poor prognosis [4].

The particularity of our case can be summarized in the exceptional gastric localization of breast metastases and the management of any emergency must call for a systematic meticulous clinical examination device by device. This will allow us to better treat our patients and to avoid all the medico-legal problems which result from it.

The disadvantage of our presentation is summed up in its rarity and the absence of a well-defined consensus for the treatment of such cases was an obstacle for evidence-based medicine. This difficulty of the case had motivated the multidisciplinary discussion which must be mandatory and which is a reminder to all concerned practitioners to share all similar cases to make the management uniform.

## Conclusions

Through our presentation, we report another case to the poor literature regarding gastric metastasis from breast cancer. The particularity of our patient lies in the rarity of clinical presentation and that emergency situation must never allow systematic clinical examinations to pass. We insist on multidisciplinary management of breast cancer and we recall that the woman has the universal right to have a screening for gynecological cancers including that of the breast.

## References

[1] Gradishar WJ, Anderson BO, Abraham J (2020). Breast Cancer, Version 3.2020, NCCN Clinical Practice Guidelines in Oncology. J Natl Compr Canc Netw.

[2] Duggan C, Dvaladze A, Rositch AF (2020). The Breast Health Global Initiative 2018 Global Summit on improving breast healthcare through resource-stratified phased implementation: methods and overview. Cancer.

[3] Emens LA, Adams S, Cimino-Mathews A (2021). Society for Immunotherapy of Cancer (SITC) clinical practice guideline on immunotherapy for the treatment of breast cancer. J Immunother Cancer.

[4] Liang Y, Zhang H, Song X, Yang Q (2020). Metastatic heterogeneity of breast cancer: molecular mechanism and potential therapeutic targets. Semin Cancer Biol.

[5] Gagnier JJ, Kienle G, Altman DG, Moher D, Sox H, Riley D (2014). The CARE guidelines: consensus-based clinical case report guideline development. J Clin Epidemiol.

[6] Wang XZ, Zeng ZY, Ye X, Sun J, Zhang ZM, Kang WM (2020). Interpretation of the development of neoadjuvant therapy for gastric cancer based on the vicissitudes of the NCCN guidelines. World J Gastrointest Oncol.

[7] Ginsburg O, Yip CH, Brooks A (2020). Breast cancer early detection: a phased approach to implementation. Cancer.

[8] Fouad TM, Barrera AM, Reuben JM (2017). Inflammatory breast cancer: a proposed conceptual shift in the UICC-AJCC TNM staging system. Lancet Oncol.

[9] Asch MJ, Wiedel PD, Habif DV (1968). Gastrointestinal metastases from crcinoma of the breast. Autopsy study and 18 cases requiring operative intervention. Arch Surg.

[10] Bolzacchini E, Nigro O, Inversini D, Giordano M, Maconi G (2021). Intestinal metastasis from breast cancer: presentation, treatment and survival from a systematic literature review. World J Clin Oncol.

[11] Apodaca-Rueda M, Chaim FH, Garcia MD (2019). Solitary pancreatic metastasis from breast cancer: case report and review of literature. Sao Paulo Med J.

[12] Noor A, Lopetegui-Lia N, Desai A, Mesologites T, Rathmann J (2020). Breast cancer metastasis masquerading as primary colon and gastric cancer: a case report. Am J Case Rep.

[13] Théraux J, Bretagnol F, Guedj N, Cazals-Hatem D, Panis Y (2009). Colorectal breast carcinoma metastasis diagnosed as an obstructive colonic primary tumor. A case report and review of the literature. Gastroenterol Clin Biol.

[14] Nehmeh WA, Derienne J, El Khoury L, Kassar S, Track-Smayra V, Noun R, Chakhtoura G (2021). A 58-year-old woman with acute gastric perforation due to metastatic ductal carcinoma 18 years following bilateral mastectomy for invasive ductal carcinoma of the breast. Am J Case Rep.

